# Psychological Well-Being, Dispositional Hope, and Contentment with Life Among Public Hospital Physicians in Türkiye’s Black Sea Region: A Cross-Sectional Study

**DOI:** 10.3390/healthcare14183053

**Published:** 2026-09-17

**Authors:** Yaşar Demir, Erhan Dağ, Mustafa Nal, Gülfer Bektaş

**Affiliations:** 1 Department of Data Science, Institute of Graduate Studies, Ondokuz Mayıs University, Samsun 55200, Türkiye; yasar.demir1@saglik.gov.tr; 2 Department of Statistics, Samsun City Hospital, Samsun 55090, Türkiye; 3 Department of Healthcare Management, Faculty of Health Sciences, Kütahya Health Sciences University, Kütahya 43100, Türkiye; erhan.dag@ksbu.edu.tr (E.D.); mustafa.nal@ksbu.edu.tr (M.N.); 4 Department of Healthcare Management, Faculty of Health Sciences, Acibadem Mehmet Ali Aydinlar University, Istanbul 34752, Türkiye; 5 Department of Healthcare Management, Graduate School of Health Sciences, Acibadem Mehmet Ali Aydinlar University, Istanbul 34752, Türkiye

**Keywords:** physicians, psychological well-being, dispositional hope, contentment with life, indirect association, cross-sectional study, Türkiye

## Abstract

Background: Physicians work under demanding conditions that may affect their psychological functioning and evaluations of life. Positive psychological resources, particularly dispositional hope, may be relevant to the association between psychological well-being and contentment with life. This study examined these constructs and their indirect association through dispositional hope among physicians working in public hospitals in Türkiye’s Black Sea region. Methods: A cross-sectional online survey was conducted between July and December 2024 with a convenience sample of 918 physicians. Validated Turkish versions of the Psychological Well-Being Scale, Dispositional Hope Scale, and Contentment with Life Assessment Scale were administered. An unadjusted indirect-association analysis was performed using PROCESS Model 4 with reliability-adjusted errors-in-variables estimation and 5000 bootstrap samples. Results: The mean scores were 37.30 ± 7.46 for psychological well-being, 45.00 ± 7.65 for dispositional hope, and 19.90 ± 5.14 for contentment with life. Psychological well-being was positively correlated with dispositional hope (*r* = 0.582) and contentment with life (*r* = 0.645), while dispositional hope was positively correlated with contentment with life (*r* = 0.465; all *p* < 0.001). Psychological well-being was positively associated with dispositional hope (β = 0.60, *B* = 0.62, *SE* = 0.08, 95% CI [0.45, 0.78], *p* < 0.001; R^2^ = 0.360). In the outcome model, psychological well-being (β = 0.46, *B* = 0.38, *SE* = 0.05, 95% CI [0.28, 0.47], *p* < 0.001) and dispositional hope (β = 0.34, *B* = 0.28, *SE* = 0.08, 95% CI [0.12, 0.44], *p* < 0.001) were positively associated with contentment with life. Together, psychological well-being and dispositional hope explained 45.5% of the variance in contentment with life (R^2^ = 0.455). The total association was statistically significant (*B* = 0.56, *SE* = 0.04, 95% CI [0.48, 0.63], *p* < 0.001), as was the indirect association through dispositional hope (β = 0.21, *B* = 0.18, Boot*SE* = 0.02, 95% bootstrap CI [0.14, 0.23]). Conclusions: Psychological well-being, dispositional hope, and contentment with life were positively associated. Hope showed a significant indirect association, but the cross-sectional, unadjusted model does not support causal inference. Longitudinal multicenter studies are needed.

## 1. Introduction

Physicians work in demanding professional environments characterized by heavy workloads, long working hours, substantial responsibility, and frequent exposure to emotionally challenging situations [1,2]. These working conditions may contribute to stress, burnout, and other psychological difficulties, which can adversely affect physicians’ psychological functioning, evaluations of their lives, and professional performance [2,3,4]. Although much of the literature concerning physicians has focused on negative outcomes such as burnout, depression, stress, and diminished well-being [4,5,6], comparatively less attention has been given to positive psychological resources that may be associated with more favorable life evaluations.

Contentment with life refers to an individual’s overall evaluation of the extent to which their life circumstances, experiences, needs, and expectations are satisfactory [7,8]. It encompasses evaluations across multiple life domains, including work, leisure, interpersonal relationships, and other non-work experiences. Previous research has demonstrated that contentment with life is associated not only with sociodemographic characteristics but also with psychological factors, particularly psychological well-being and dispositional hope [9,10,11]. Examining these positive psychological constructs together may therefore contribute to a more comprehensive understanding of physicians’ evaluations of their lives.

According to Snyder’s Hope Theory, dispositional hope is a goal-directed psychological resource comprising two related components: agency thinking, which reflects the motivation to pursue desired goals, and pathways thinking, which refers to the perceived ability to identify workable routes toward achieving those goals [12,13]. Hope may be particularly relevant to physicians because maintaining motivation and identifying alternative pathways may help them respond adaptively to professional demands and obstacles. Previous studies have shown that dispositional hope is positively associated with emotional functioning, psychological well-being, coping, resilience, and contentment with life [14,15,16,17].

In the present study, psychological well-being was operationalized using the eight-item Psychological Well-Being Scale developed by Diener et al. and adapted into Turkish by Telef [18,19]. Consistent with this measure, psychological well-being is conceptualized as a broad evaluation of positive psychological functioning that includes having a purposeful and meaningful life, supportive and rewarding relationships, engagement in daily activities, perceived competence, self-respect, optimism, and a sense of contributing to the well-being of others. This conceptualization differs from Ryff’s six-dimensional model and directly corresponds to the construct assessed by the instrument used in this study. Higher psychological well-being has been associated with more adaptive functioning and more favorable evaluations of life, making it particularly relevant in occupational groups exposed to sustained psychological and organizational demands [20,21,22,23].

Psychological well-being may be associated with contentment with life both directly and indirectly through dispositional hope [21,24,25]. Psychological well-being, as operationalized by Diener et al., encompasses meaning and purpose, competence, optimism, supportive relationships, and engagement in daily activities [18,19]. From the perspective of Hope Theory, these positive-functioning characteristics may be relevant to agency thinking—the motivation to pursue goals—and pathways thinking—the perceived capacity to generate alternative routes toward goal attainment [13,26]. Dispositional hope may, in turn, be associated with more favorable evaluations of life by supporting goal-directed thinking and adaptive responses to obstacles [13,24,25,26]. On this theoretical basis, dispositional hope was specified as an intervening variable in the association between psychological well-being and contentment with life. Nevertheless, hope may also operate as an antecedent or a parallel positive psychological resource. Because all variables were measured simultaneously, the proposed ordering represents a theoretically specified cross-sectional model and does not establish temporal precedence or causal mediation [27,28].

**Figure 1:**
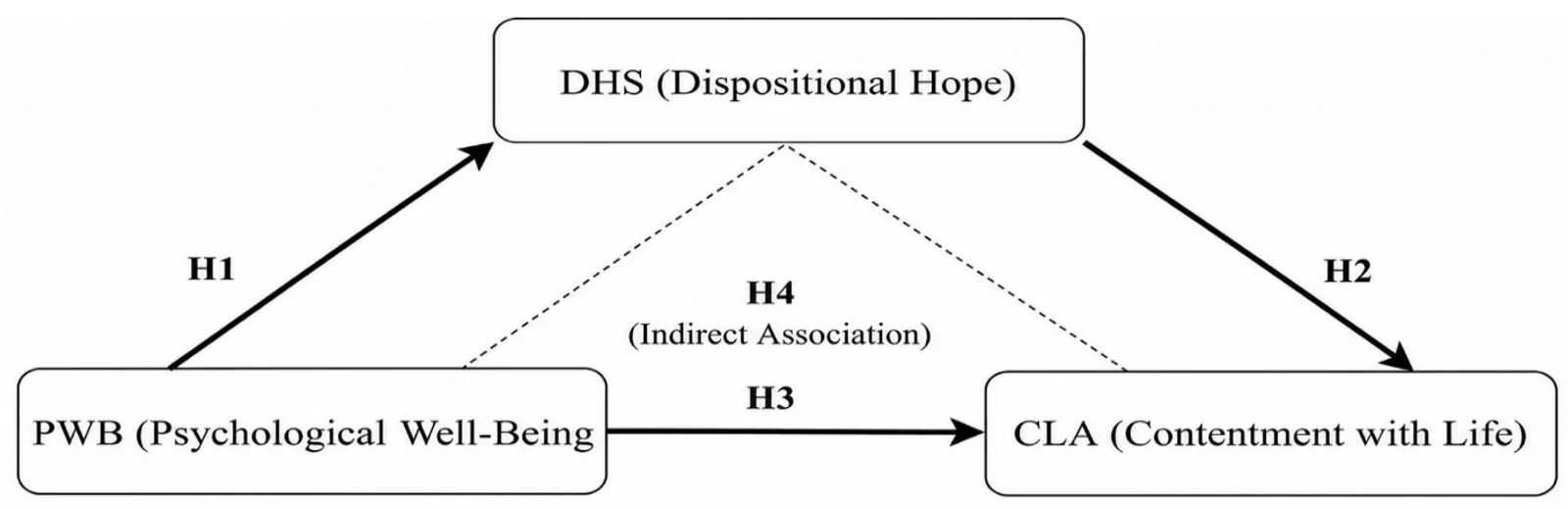
Theoretically specified cross-sectional model of the indirect association between psychological well-being and contentment with life through dispositional hope.

Within the Turkish healthcare system, physicians work under conditions characterized by high service demand and a physician-to-population ratio below the OECD average [29,30]. Previous research involving physicians in Türkiye has predominantly examined negative psychological outcomes, including burnout, depression, anxiety, and stress, whereas relatively few studies have considered psychological well-being, dispositional hope, and contentment with life within the same model. Evidence regarding the indirect association between psychological well-being and contentment with life through dispositional hope is especially limited in physician populations. Therefore, this study aimed to examine psychological well-being, dispositional hope, and contentment with life among physicians working in public hospitals in Türkiye’s Black Sea region and to test whether psychological well-being was indirectly associated with contentment with life through dispositional hope in a cross-sectional model (Figure 1). Accordingly, the principal contribution of the study is to examine whether this theoretically specified pattern of associations is present in this particular physician population, rather than to establish a causal psychological mechanism.

Research hypotheses

**H1.**  *Psychological well-being is positively associated with dispositional hope*.

**H2.**  *Dispositional hope is positively associated with contentment with life*.

**H3.**  *Psychological well-being is positively associated with contentment with life*.

**H4.**  *Psychological well-being is indirectly associated with contentment with life through dispositional hope in the theoretically specified cross-sectional model*.

## 2. Materials and Methods

### 2.1. Research Design

We used a cross-sectional and correlational design, following the STROBE checklist [31,32].

### 2.2. Participants and Procedures

This cross-sectional study included a convenience sample of physicians employed in public hospitals in Türkiye’s Black Sea region. Data were collected between July and December 2024 using an online, self-administered questionnaire administered through Google Forms. The online method was selected because the target population was geographically dispersed and physicians had demanding work schedules.

The researchers contacted chief clinicians at accessible public hospitals and asked them to distribute the study invitation to potentially eligible physicians through professional WhatsApp groups. Participation was voluntary and based on electronic informed consent. Because the invitation was distributed indirectly through professional WhatsApp groups, the researchers could not determine the exact number of physicians who received or viewed the invitation. Consequently, a response rate could not be calculated. Hospital-identifying information was not collected, and the number of participating hospitals and the distribution of participants across hospitals could not be determined.

The inclusion criteria were: (1) being a physician, (2) working in a public hospital in Türkiye’s Black Sea region during the data-collection period, and (3) providing electronic informed consent. Individuals who did not provide electronic informed consent could not proceed to the questionnaire. No additional exclusion criteria were applied.

Before accessing the questionnaire, participants were informed about the purpose and scope of the study, the voluntary nature of participation, anonymity and confidentiality procedures, and their right to withdraw from the study without consequences. The first page of the online form contained the electronic informed consent statement, “I agree to participate in the study.” Only individuals who selected “I agree” were permitted to proceed to the questionnaire.

To improve data quality, all relevant survey items were marked as required, thereby preventing incomplete submissions. The “Limit to 1 response” setting was also enabled to reduce the likelihood of duplicate participation. Although this setting required participants to sign in with a Google account, the form was not configured to collect email addresses. Therefore, no email addresses or other direct identifiers were stored or exported, and responses were analyzed anonymously.

A total of 1000 individuals accessed the electronic consent screen. Of these, 82 selected “I do not consent” and were not permitted to proceed to the questionnaire. The remaining 918 physicians provided electronic informed consent and completed the survey; all were included in the analytical sample. No participants were excluded from the analytical dataset after completing the questionnaire.

An a priori power analysis was conducted using G*Power version 3.1.9.7 for a general multiple regression model with two predictors [33,34]. Assuming an alpha level of 0.05, statistical power of 0.95, and a medium effect size of *f*^2^ = 0.15, the minimum required sample size was estimated as 107. The final sample of 918 exceeded this regression-based estimate. However, this calculation was not specifically designed to determine statistical power for the bootstrap-based indirect association, and no formal a priori power analysis was conducted specifically for the indirect association.

Convenience sampling, voluntary online participation, recruitment through professional WhatsApp groups, and restriction of the sample to physicians working in public hospitals in one geographical region may have introduced self-selection bias and limited the representativeness and generalizability of the findings. Furthermore, because hospital-identifying information was not collected, the potential clustering of physicians within hospitals could not be evaluated using cluster-robust or multilevel methods. This may have affected the estimated precision of the associations and should be considered when interpreting the findings. Finally, because all variables were measured simultaneously using self-report instruments, temporal ordering could not be established. The findings were therefore interpreted as cross-sectional direct and indirect associations rather than evidence of causal mediation [27,28].

Ethical approval for this study was obtained from the Kütahya Health Sciences University Non-Interventional Clinical Research Ethics Committee (decision no. 2024/07-17; approval date: 17 May 2024). All procedures were conducted in accordance with the ethical principles of the Declaration of Helsinki. Participation was voluntary, and electronic informed consent was obtained from all participants before they accessed the questionnaire.

### 2.3. Measures

#### 2.3.1. Personal Information Form

The personal information form included age, gender, marital status, professional title, and professional experience.

#### 2.3.2. Psychological Well-Being Scale (PWBS)

Psychological well-being was assessed using the eight-item Psychological Well-Being Scale developed by Diener et al. [18], and adapted into Turkish by Telef [19]. The scale assesses broad positive psychological functioning, including meaning and purpose, supportive relationships, engagement in daily activities, perceived competence, self-respect, optimism, and social contribution. Thus, the instrument reflects a broader flourishing-oriented conceptualization of psychological well-being rather than Ryff’s six-dimensional model. Each item is rated on a seven-point Likert-type scale ranging from 1 (“strongly disagree”) to 7 (“strongly agree”). Total scores range from 8 to 56, with higher scores indicating greater psychological well-being. Cronbach’s alpha coefficients of 0.87 and 0.89 were previously reported for the original and Turkish versions, respectively. In the present study, Cronbach’s alpha was 0.87. A confirmatory factor analysis was conducted to examine the single-factor structure of the scale in the present physician sample. The model demonstrated an acceptable fit χ^2^(20) = 97.30, *p* < 0.001, χ^2^/df = 4.87, RMSEA = 0.071, CFI = 0.90, and TLI = 0.93.

#### 2.3.3. The Dispositional Hope Scale (DHS)

Dispositional hope was assessed using the Dispositional Hope Scale developed by Snyder et al. [26], and adapted into Turkish by Tarhan and Bacanlı [35]. The original scale comprises 12 items: four agency items, four pathways items, and four distractor items. In the present study, the four distractor items were excluded from scoring and statistical analyses; therefore, only the eight substantive items—four measuring agency thinking and four measuring pathways thinking—were considered. Each substantive item is rated on an eight-point scale ranging from 1 (“definitely false”) to 8 (“definitely true”). The total dispositional hope score was calculated by summing the eight substantive agency and pathways items and ranged from 8 to 64, with higher scores indicating greater dispositional hope. Cronbach’s alpha coefficients of 0.86 and 0.83 were reported for the original and Turkish versions, respectively. In the present study, Cronbach’s alpha for the eight-item total score was 0.83.

A confirmatory factor analysis was conducted to examine the correlated two-factor structure of the eight scored items in the present physician sample. The four distractor items were not included in the confirmatory factor analysis because they are not indicators of the agency or pathways constructs. The correlated two-factor model demonstrated an acceptable fit: χ^2^(19) = 51.15, *p* < 0.001, χ^2^/df = 2.69, RMSEA = 0.043, CFI = 0.91, and TLI = 0.92. These findings supported the agency and pathways structure of the DHS in the present sample.

The total DHS score was used in the primary indirect-association analysis because the prespecified research model focused on global dispositional hope rather than the distinct contributions of agency and pathways thinking. Although the two-factor structure was examined through confirmatory factor analysis, separate indirect-association analyses for the two subscales were not included in the primary model.

#### 2.3.4. Contentment with Life Scale (CLAS)

Contentment with life was assessed using the five-item Contentment with Life Assessment Scale developed by Lavallee et al. [8], and adapted into Turkish by Akın and Yalnız [7]. Each item is rated on a seven-point Likert-type scale ranging from 1 (“strongly disagree”) to 7 (“strongly agree”). The third and fourth items are reverse-scored. In the present study, the observed scores ranged from 5 to 35. Cronbach’s alpha coefficients of 0.85 and 0.73 were reported for the original and Turkish versions, respectively. Cronbach’s alpha was 0.79 in the present study. A confirmatory factor analysis was conducted to examine the single-factor structure of the CLAS in the present physician sample. The model demonstrated an acceptable fit: χ^2^(5) = 19.77, *p* < 0.01, χ^2^/df = 3.95, RMSEA = 0.069, CFI = 0.94, and TLI = 0.96.

### 2.4. Data Analysis

The data were analyzed using IBM SPSS Statistics version 26.0 (IBM Corp., Armonk, NY, USA), Hayes’s PROCESS macro version 5.0, and Jamovi version 2.6.17. Descriptive statistics, including means, standard deviations, observed minimum and maximum values, skewness, and kurtosis, were calculated for the study variables. Pearson correlation coefficients were used to examine the bivariate associations among psychological well-being, dispositional hope, and contentment with life.

Confirmatory factor analyses were conducted to examine the prespecified factor structures of the three scales in the present physician sample. Model fit was evaluated using the chi-square statistic, chi-square-to-degrees-of-freedom ratio (χ^2^/df), root mean square error of approximation (RMSEA), comparative fit index (CFI), and Tucker–Lewis index (TLI). Internal consistency reliability was evaluated using Cronbach’s alpha.

Given the cross-sectional design, the indirect-association analysis was conducted and interpreted within an associational framework rather than as evidence of temporal or causal mediation. Psychological well-being was specified as the independent variable, dispositional hope as the intervening variable, and contentment with life as the dependent variable using Model 4 of Hayes’s PROCESS macro. This ordering was theoretically specified; however, because all variables were measured simultaneously, temporal precedence could not be established [27,28]. Reliability-adjusted estimates were obtained using the errors-in-variables (EIV) approach described by Hayes et al. [36]. The Cronbach’s alpha coefficients obtained in the present sample—0.87 for psychological well-being, 0.83 for dispositional hope, and 0.79 for contentment with life—were entered as the reliability estimates. The EIV procedure was used to reduce potential attenuation of regression coefficients associated with random measurement error in the observed scale scores. Conventional PROCESS estimates without correction for measurement unreliability were not calculated; therefore, conventional and EIV-adjusted estimates were not compared.

The total association between psychological well-being and contentment with life, the direct association after accounting for dispositional hope, and the indirect association through dispositional hope were estimated. The component paths from psychological well-being to dispositional hope and from dispositional hope to contentment with life after accounting for psychological well-being were also examined. Unstandardized coefficients, standard errors, standardized coefficients, and 95% confidence intervals were reported where applicable. The indirect association was estimated using 5000 bootstrap samples and was considered statistically significant when its 95% bootstrap confidence interval did not include zero.

The model was estimated without sociodemographic covariates. Therefore, the reported coefficients represent unadjusted, reliability-adjusted cross-sectional associations. Potential confounding by age, gender, marital status, professional title, professional experience, perceived income, and other unmeasured occupational or organizational characteristics cannot be excluded. No covariate-adjusted sensitivity analysis was conducted.

Before conducting the regression analyses, the distributions of the study variables were examined using skewness and kurtosis values [37,38]. Linearity and homoscedasticity were assessed using plots of residuals against fitted values, and the distribution of the residuals was evaluated using Q–Q plots. Multicollinearity was assessed using tolerance and variance inflation factor (VIF) statistics. The Durbin–Watson statistic was used only to assess autocorrelation of the regression residuals and was not interpreted as a measure of within-hospital dependence [37]. Because hospital-identifying information was not collected, potential clustering of physicians within hospitals could not be assessed using cluster-robust or multilevel methods. All statistical tests were two-tailed, and a *p* value < 0.05 was considered statistically significant.

## 3. Results

### 3.1. Common Method Bias

The potential for common method bias was preliminarily examined using Harman’s single-factor test based on an unrotated exploratory factor analysis [39]. The first unrotated factor accounted for 41.85% of the total variance, indicating that no single factor dominated the variance in the measured items. However, Harman’s single-factor test is a limited diagnostic procedure and cannot establish the absence of common method bias. Therefore, the possibility that the observed associations were affected by shared method variance could not be ruled out and was considered when interpreting the findings. Procedural measures intended to reduce evaluation apprehension and response bias included emphasizing the anonymity and confidentiality of responses and informing participants that there were no correct or incorrect answers.

### 3.2. Participant Characteristics

The mean age of the physicians was 40.43 ± 9.69 (min. 23–max. 65) and the mean number of years of professional experience was 14.71 ± 10.19 (min. 1–max. 40). Among the physicians who participated in the study, 76.10% were married and 47.80% were specialist physicians. Other information about the physicians is shown in Table 1.

Descriptive statistics, including mean, standard deviation, skewness, and kurtosis values, are presented in Table 2. The skewness and kurtosis coefficients ranged between ± 2, suggesting that the study variables did not show substantial deviations from normality [40].

The total scale scores were M = 37.30 for psychological well-being (PWB, M = 45.00 for dispositional hope scale (DHS), and M = 19.90 for contentment with life (CLAS) (Table 2).

Regarding bivariate correlations, psychological well-being was positively and significantly correlated with dispositional hope and contentment with life (*r* = 0.582, *p* < 0.001; *r* = 0.645, *p* < 0.001, respectively). Dispositional hope was positively and significantly correlated with contentment with life (*r* = 0.465, *p* < 0.001) (Table 2).

Although the bivariate correlations among the study variables were moderate, multicollinearity diagnostics were examined before conducting the regression-based indirect-association analysis. In the outcome model, psychological well-being and dispositional hope were included simultaneously as predictors of contentment with life. The tolerance value was 0.661, which was above the recommended minimum of 0.10, and the corresponding variance inflation factor was 1.512, which was below the conservative threshold of 5.00. These findings indicated no problematic multicollinearity between psychological well-being and dispositional hope.

The Durbin–Watson statistic was 2.10, indicating no substantial autocorrelation in the regression residuals. This statistic was used only to assess residual autocorrelation and was not interpreted as a test of dependence among physicians working within the same hospital. Because hospital-identifying information was not collected, potential within-hospital clustering could not be evaluated. Visual examination of the residual-versus-fitted plots and Q–Q plots did not indicate serious violations of linearity, homoscedasticity, or residual-distribution assumptions.

### 3.3. Indirect Association Analysis Results

The indirect-association analysis was conducted using Model 4 of the PROCESS macro version 5.0 with reliability-adjusted errors-in-variables (EIV) estimation. No sociodemographic covariates were included; therefore, the reported coefficients represent unadjusted, reliability-adjusted associations. Given the cross-sectional design, the model was interpreted within an associational framework rather than as evidence of temporal or causal mediation.

Psychological well-being was found to be positively associated with dispositional hope (β = 0.60, *B* = 0.62, *SE* = 0.08, 95% CI [0.45, 0.78], *p* < 0.001). The mediating variable model explained 36.0% of the variance in dispositional hope (R^2^ = 0.360).

In the final model, when dispositional hope was controlled for, the positive relationship between psychological well-being and contentment with life persisted (β = 0.46, *B* = 0.38, *SE* = 0.05, 95% CI [0.28, 0.47], *p* < 0.001). When psychological well-being was controlled for, dispositional hope was also found to be positively associated contentment with life (β = 0.34, *B* = 0.28, *SE* = 0.08, 95% CI [0.12, 0.44], *p* < 0.001). Psychological well-being and dispositional hope together explained 45.5% of the variance in contentment with life (R^2^ = 0.455).

The overall relationship between psychological well-being and contentment with life is positive and statistically significant (*B* = 0.56, *SE* = 0.04, 95% CI [0.48, 0.63], *p* < 0.001). After dispositional hope was included in the final model, the direct association between psychological well-being and contentment with life remained statistically significant.

**Figure 2:**
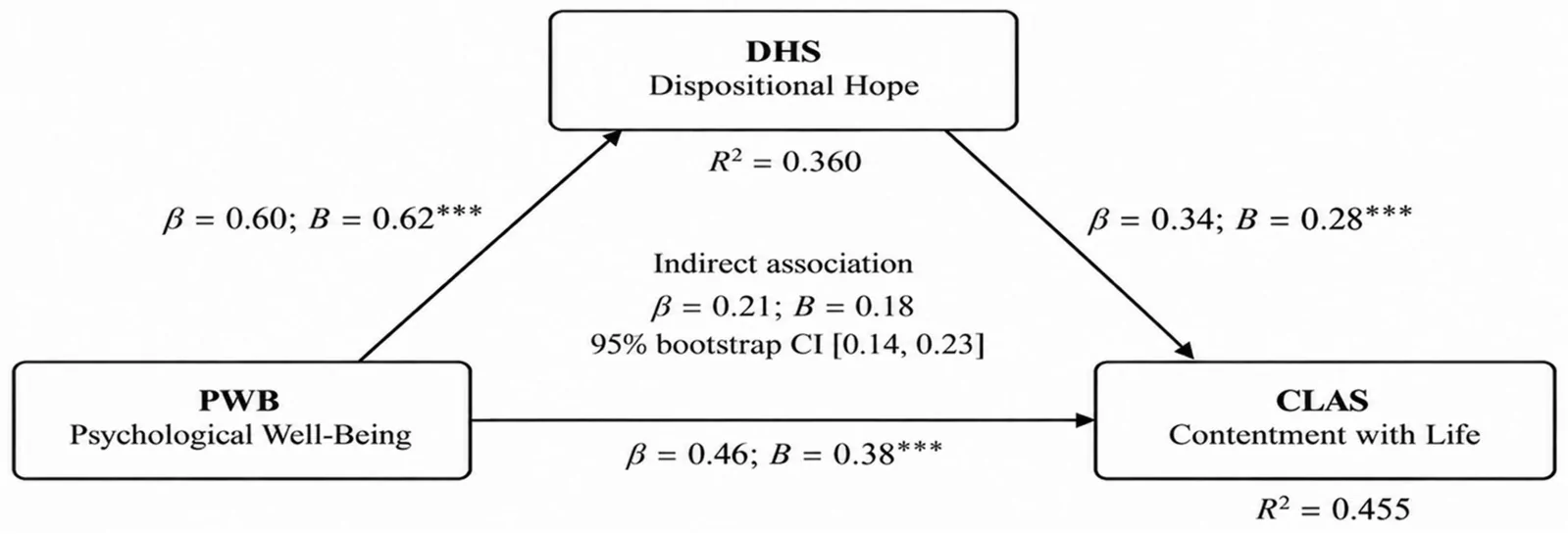
Indirect Association Model of Psychological Well-Being, Dispositional Hope, and Contentment With Life. Note: *** *p* < 0.001.

The indirect association between psychological well-being and contentment with life through dispositional hope was statistically significant (β = 0.21, *B* = 0.18, Boot*SE* = 0.02, 95% bootstrap CI [0.14, 0.23]). The proportion of the indirect relationship to the total association was 32.14% (0.18/0.56 × 100). This proportion is presented solely as a descriptive breakdown of the observed relationship and should not be interpreted as the extent to which dispositional hope causally explain the association in question. Because sociodemographic covariates were not included in the model and all variables were measured simultaneously, the findings do not reveal the independent effects of covariates, temporal sequence, or causal mediation (Table 3 and Figure 2).

Descriptive proportion of the total association = indirect association/total association × 100 (0.18/0.56 × 100 = 32.14%).

## 4. Discussion

This study examined the associations among psychological well-being, dispositional hope, and contentment with life in physicians working in public hospitals in Türkiye’s Black Sea region. Psychological well-being was positively associated with both dispositional hope and contentment with life, and dispositional hope was positively associated with contentment with life after accounting for psychological well-being. The analysis also identified a statistically significant indirect association between psychological well-being and contentment with life through dispositional hope. These findings provide cross-sectional support for the theoretically specified pattern of associations; however, they do not establish temporal ordering or causal mediation.

The mean scores observed for psychological well-being, dispositional hope, and contentment with life provide a descriptive profile of the study sample. They should not be interpreted as indicating low, moderate, or high levels because the instruments do not provide established clinical cutoff values for such classifications. The results should also be considered within the socioeconomic and occupational context of the participating physicians. In particular, 92.2% reported that their income was lower than their expenditure. The “income greater than expenditure” option was available in the questionnaire but was not selected by any participant. This distribution may reflect perceived financial strain under the economic conditions prevailing during the data-collection period. Nevertheless, the determinants of perceived income were not examined, and economic conditions cannot be considered demonstrated explanations for the observed psychological associations. Payment structures within the Turkish healthcare system also vary according to institutional and service-related characteristics [41], but the present study did not directly assess payment conditions or their relationship with contentment with life.

Other occupational conditions may also provide context for interpreting the findings. Previous studies in Türkiye have reported associations of workload and occupational stress with burnout, job satisfaction, and intention to leave among healthcare professionals [42,43]. Workplace violence, malpractice concerns, professional working conditions, and socioeconomic factors have also been discussed in relation to physicians’ professional experiences, specialty preferences, and migration decisions [44,45]. These factors were not directly measured in the present study and should not be treated as explanations for the observed associations. Rather, they identify potentially relevant contextual variables that should be incorporated into future research.

The positive association between psychological well-being and contentment with life is consistent with previous evidence showing that positive psychological functioning is related to more favorable evaluations of life [11,22,23]. In the present study, psychological well-being was operationalized using the eight-item measure developed by Diener et al. and adapted into Turkish by Telef. Accordingly, it reflects broad positive functioning involving meaning and purpose, supportive relationships, engagement in daily activities, perceived competence, self-respect, optimism, and social contribution. This conceptualization, rather than Ryff’s six-dimensional model, corresponds to the instrument used in the study. These aspects of positive functioning may be particularly relevant to physicians, whose professional responsibilities require sustained decision-making, emotional functioning, interpersonal communication, and adaptation to challenging working conditions. Nevertheless, the observed association does not demonstrate that psychological well-being causes greater contentment with life.

Dispositional hope was also positively associated with contentment with life. This finding is consistent with previous studies linking hope with subjective well-being, psychological functioning, and life satisfaction [16,17,21]. According to Hope Theory, dispositional hope comprises agency thinking, which refers to the motivation to pursue goals, and pathways thinking, which refers to the perceived capacity to identify workable routes toward goal attainment [13]. These characteristics may help individuals maintain goal-directed thinking and identify alternative strategies when encountering obstacles. However, the present analysis used the total DHS score and did not examine separate indirect associations for agency and pathways. Therefore, no conclusions can be drawn regarding whether these two dimensions have different relationships with psychological well-being or contentment with life.

The significant indirect association through dispositional hope is theoretically consistent with research indicating that hope may occupy an important position in associations between positive psychological characteristics and evaluations of life. Hatun and Kurtça (2026) reported longitudinal associations involving perceived stress, hope, and life satisfaction [24], while Tran et al. (2024) examined hope in relation to self-compassion, psychological well-being, and life satisfaction [25]. Related associations between dispositional hope and subjective well-being have also been reported [16,21]. The present study extends this literature by identifying a comparable statistical pattern in a sample of physicians working in public hospitals in Türkiye’s Black Sea region.

The ratio of the indirect association to the total association was approximately 32.14%. This value is presented solely as a descriptive decomposition of the estimated associations and should not be interpreted as indicating that dispositional hope causally explained approximately one-third of the relationship. The remaining direct association suggests that the statistical pattern was not entirely represented by dispositional hope and that other psychological, socioeconomic, occupational, and organizational characteristics may also be relevant. Moreover, because no sociodemographic covariates were included in the model, residual confounding cannot be excluded.

The findings are also relevant to the broader literature on positive psychological resources among healthcare professionals. Psychological resilience has been associated with lower burnout among physicians, while diminished psychological well-being remains an important concern in physician populations [46]. Associations between positive psychological resources and professional quality of life have also been reported among other healthcare professionals [47]. The present results support further examination of positive psychological resources alongside traditional indicators of distress, such as burnout, depression, anxiety, and occupational stress, but they do not demonstrate the effectiveness of interventions targeting these resources.

Structured peer support, confidential psychological counseling, mentoring, realistic goal-setting approaches, and programs addressing adaptive coping may be considered potential directions for future intervention research. Their feasibility and effectiveness should be evaluated using longitudinal, experimental, or quasi-experimental designs before specific implementation recommendations are made. Such individual-level approaches should complement, rather than replace, organizational and structural measures addressing workload, working hours, work–life balance, workplace safety, professional autonomy, economic conditions, and institutional support.

Overall, the study identified theoretically meaningful associations among psychological well-being, dispositional hope, and contentment with life in the study sample. Its contribution lies in demonstrating that this specified pattern is present in physicians working in public hospitals in one region of Türkiye, rather than in establishing a causal psychological process. Future multicenter and longitudinal studies should examine these associations across different geographical regions and healthcare settings while incorporating sociodemographic and occupational covariates, organizational characteristics, hospital-level clustering, and separate agency and pathways analyses.

### 4.1. Theoretical Implications

The present findings contribute to the literature on positive psychological resources by providing evidence of positive associations among psychological well-being, dispositional hope, and contentment with life in physicians working in public hospitals in Türkiye’s Black Sea region. Psychological well-being was associated with contentment with life both directly and indirectly through dispositional hope. This pattern suggests that dispositional hope may represent a potential explanatory pathway or intervening correlate in the association between psychological well-being and contentment with life. However, it should not be interpreted as evidence that hope constitutes a temporal or causal psychological mechanism.

The ratio of the indirect association to the total association was approximately 32%. This value is presented solely as a descriptive decomposition of the estimated associations and does not indicate that dispositional hope causally explained 32% of the relationship between psychological well-being and contentment with life. The remaining direct association suggests that the statistical pattern was not entirely represented by dispositional hope and that other psychological, occupational, social, and organizational characteristics may also be relevant. Moreover, because the model was not adjusted for sociodemographic or occupational covariates, residual confounding cannot be excluded.

The observed pattern is theoretically consistent with Hope Theory, which conceptualizes dispositional hope as comprising agency thinking—the motivation to pursue goals—and pathways thinking—the perceived capacity to identify workable routes toward goal attainment. Positive psychological functioning may be associated with physicians’ capacity to maintain goal-directed motivation and identify alternative pathways when confronting professional challenges. These characteristics may, in turn, be associated with more favorable evaluations of life. Nevertheless, dispositional hope could also operate as an antecedent to psychological well-being or as a parallel positive psychological resource. Because all variables were measured simultaneously, the proposed ordering represents one theoretically specified cross-sectional model rather than an empirically established temporal sequence. Longitudinal studies with temporally separated measurements and experimental research are required to evaluate the direction and potential causal nature of these associations.

### 4.2. Practical Implications

The observed associations suggest that physicians’ psychological well-being and dispositional hope may be relevant considerations within broader organizational strategies intended to support physicians’ well-being. However, the present study did not evaluate any organizational or psychological intervention. Therefore, the findings should not be interpreted as evidence that strengthening psychological well-being or dispositional hope would lead to improvements in contentment with life.

Structured peer support, confidential psychological counseling, mentoring, goal-setting programs, and approaches addressing agency and pathways thinking may be considered potential directions for future intervention research. The feasibility, acceptability, and effectiveness of these approaches should be evaluated through appropriately designed longitudinal, experimental, or quasi-experimental studies before specific implementation recommendations can be made.

Importantly, the findings do not imply that responsibility for physicians’ well-being rests primarily with individual psychological resources. Workload, working hours, work–life balance, organizational climate, economic conditions, workplace safety, professional autonomy, and institutional support were not directly examined in the present study. Moreover, the principal model was not adjusted for sociodemographic, occupational, or organizational covariates. Therefore, the observed associations may partly reflect unmeasured individual and contextual factors.

Future research should adopt a multilevel perspective that examines individual psychological resources alongside organizational working conditions and broader health-system factors. Longitudinal and intervention studies are needed to determine whether changes in psychological well-being or dispositional hope precede changes in contentment with life and whether individual-level support provides additional benefits when combined with organizational and structural improvements. Because the present sample was limited to physicians working in public hospitals in Türkiye’s Black Sea region, the relevance of these potential approaches should also be examined in other geographical regions and healthcare settings.

### 4.3. Limitations

Several limitations should be considered when interpreting the findings. First, the cross-sectional design precludes conclusions regarding temporal ordering or causality. Although a statistically significant indirect association was observed, it cannot be determined whether psychological well-being precedes dispositional hope, whether hope precedes psychological well-being, or whether both constructs operate as parallel psychological resources. Accordingly, the theoretically specified model should not be interpreted as evidence of temporal or causal mediation. Longitudinal studies with temporally separated measurements and experimental research are needed to evaluate the direction and potential causal nature of these associations.

Second, all study variables were assessed using self-report instruments administered within the same survey. This may have introduced social desirability, response, and common method biases. The first unrotated factor in Harman’s single-factor test accounted for 41.85% of the total variance, indicating that no single factor dominated the variance. Nevertheless, Harman’s test is a limited diagnostic procedure and cannot establish the absence of common method bias. Therefore, shared method variance may have contributed to the observed associations.

Third, participants were recruited through convenience sampling and professional WhatsApp groups, and participation was voluntary. Because the invitation was distributed indirectly, the exact number of physicians who received or viewed it could not be determined, and a response rate could not be calculated. These recruitment procedures may have introduced self-selection bias. Hospital-identifying information was not collected; therefore, the number of participating hospitals, the distribution of participants across hospitals, and the extent of institutional clustering could not be determined. Consequently, within-hospital dependence could not be assessed using intraclass correlation coefficients, cluster-robust standard errors, or multilevel models. This limitation may have affected the estimated precision of the associations.

Fourth, the sample was restricted to physicians working in public hospitals in Türkiye’s Black Sea region. The findings should therefore not be generalized without caution to physicians working in other geographical regions, university hospitals, private healthcare institutions, or the overall physician population in Türkiye.

Fifth, the principal model was estimated without sociodemographic covariates. Although age, gender, marital status, professional title, professional experience, and perceived income were collected, these variables were not included in the indirect-association model, and no covariate-adjusted sensitivity analysis was conducted. In addition, potentially relevant occupational and organizational characteristics—including medical specialty, workload, working hours, work–life balance, workplace violence, professional autonomy, organizational climate, and institutional support—were not measured or incorporated into the model. Residual confounding by measured and unmeasured sociodemographic, occupational, and organizational factors therefore cannot be excluded. The findings should consequently be interpreted as unadjusted cross-sectional associations rather than covariate-independent effects.

Sixth, the reported coefficients were estimated using reliability-adjusted EIV regression. Conventional PROCESS estimates without correction for measurement unreliability were not calculated; therefore, the extent to which the magnitude of the findings depended on the EIV correction could not be evaluated. Future studies should report conventional and reliability-adjusted estimates together to assess the robustness of the substantive conclusions.

Seventh, although confirmatory factor analyses were conducted to examine the prespecified structures of the three scales, the principal indirect-association model used the total DHS score. Separate indirect-association analyses were not conducted for the agency and pathways subscales. The use of the total score may therefore have obscured potentially different associations involving the motivational and route-generating components of dispositional hope.

Eighth, the confirmatory factor analyses were conducted separately for the PWB, DHS, and CLAS. Therefore, these analyses support the prespecified internal factor structure of each instrument within the present physician sample but do not directly demonstrate that psychological well-being, dispositional hope, and contentment with life are empirically distinct constructs. A joint measurement model incorporating all three constructs was not examined. Future studies should evaluate their empirical distinctiveness using a joint measurement model and appropriate assessments of discriminant validity.

Finally, the reported G*Power calculation was based on a general multiple regression model with two predictors and was not specifically designed to assess statistical power for the bootstrap-based indirect association. No formal a priori power analysis was conducted specifically for the indirect association. Although the sample was large and the bootstrap confidence interval indicated a statistically precise indirect estimate, this does not replace an analysis-specific a priori power assessment. Future multicenter and longitudinal studies should incorporate prespecified power analyses for indirect associations, relevant covariates, hospital-level clustering, and occupational and organizational characteristics.

## 5. Conclusions

This study identified positive associations among psychological well-being, dispositional hope, and contentment with life in physicians working in public hospitals in Türkiye’s Black Sea region. Psychological well-being was associated with contentment with life both directly and indirectly through dispositional hope. The indirect-to-total association ratio was approximately 32%; however, this value represents only a descriptive decomposition and should not be interpreted as the proportion of the association causally explained by dispositional hope.

Because the study was cross-sectional and the model was not adjusted for sociodemographic, occupational, or organizational covariates, the findings do not establish temporal ordering, causal mediation, or covariate-independent effects. Dispositional hope should therefore be regarded as a potential explanatory correlate rather than a demonstrated psychological mechanism. The findings also do not establish the effectiveness of interventions targeting psychological well-being or hope.

Future multicenter and longitudinal studies should examine whether these associations persist across different regions and healthcare settings and whether changes in psychological well-being or dispositional hope precede changes in contentment with life. Future research should also incorporate relevant sociodemographic and occupational covariates, hospital-level clustering, and organizational factors such as workload, working hours, work–life balance, workplace safety, professional autonomy, and institutional support. Intervention studies are needed before specific physician-support strategies can be recommended.

## Figures and Tables

**Table 1:** Socio-Demographic Characteristics of Participants.

Variables	*n* (918)	%
Age		
23–29	189	20.5
30–39	257	27.9
40–49	295	32.1
50–65	177	19.5
Gender		
Female	416	45.3
Male	502	54.7
Marital status		
Married	699	76.1
Single	219	23.9
Title		
Prof.; Assoc. Prof.; Assist. Prof.	211	23.0
Specialist Physician	438	47.8
Physician	105	11.4
Assistant Physician	164	17.8
Professional experience		
0–15 years	479	52.1
≥16 years	439	47.9
Perceived income *		
Income less than expenditure	847	92.2
Income equal to expenditure	71	7.8

* Note. The perceived-income question included all three response options. The “income greater than expenditure” option was available in the questionnaire but was not selected by any participant.

**Table 2:** Descriptive Statistics and Correlation Coefficients of Variables.

Variables	Items	Min–Max	M	SD	Skewness	Kurtosis	1	2	3
1. Psychological Well-Being (PWB)	8	8–56	37.30	7.46	0.05	−0.73	1	0.582 ***	0.645 ***
2. Dispositional Hope (DHS)	8	8–64	45.00	7.65	−0.25	−0.86		1	0.465 ***
3. Contentment with Life (CLAS)	5	5–35	19.90	5.14	−0.21	−1.09			1

Note. *** *p* < 0.001 (two-tailed).

**Table 3:** Indirect Association Analysis Results.

Model and Path	β	*B*	*SE*/Boot*SE*	95% CI	*p*
Mediator model: Outcome = dispositional hope (R^2^ = 0.360)					
Psychological well-being → Dispositional hope (a path)	0.60	0.62	0.08	[0.45, 0.78]	<0.001
Outcome model: Outcome = contentment with life (R^2^ = 0.455)					
Psychological well-being → Contentment with life (c′ path)	0.46	0.38	0.05	[0.28, 0.47]	<0.001
Dispositional hope → Contentment with life (b path)	0.34	0.28	0.08	[0.12, 0.44]	<0.001
Total-association model					
Psychological well-being → Contentment with life (c path)	—	0.56	0.04	[0.48, 0.63]	<0.001
Indirect association					
Psychological well-being → Dispositional hope → Contentment with life (ab)	0.21	0.18	0.02	[0.14, 0.23]	—

Note. No sociodemographic covariates were included. β = standardized coefficient; *B* = unstandardized coefficient; *SE* = standard error; Boot*SE* = bootstrap standard error; CI = confidence interval.

## Data Availability

The datasets used and/or analyzed during the current study are not publicly available due to ethical and privacy restrictions but are available from the corresponding author upon reasonable request.
